# Special Issue: Semiconductor Heterostructures (with Quantum Wells, Quantum Dots and Superlattices)

**DOI:** 10.3390/nano12101685

**Published:** 2022-05-15

**Authors:** Valentin Jmerik

**Affiliations:** Centre of Nanoheterostructure Physics, Ioffe Institute, 26 Politekhnicheskaya, 194021 St. Petersburg, Russia; jmerik@pls.ioffe.ru

Semiconductor heterostructures form the basis of modern electronics and optoelectronics, and the study of physical phenomena in them, along with the development of technological methods for their manufacture, is actively carried out all over the world to ensure progress in the output parameters of devices. The most important trends in the development of semiconductor heterostructures are both the development of new systems of materials and the continuous decrease in the size of active regions of various types of heterostructures, including superlattices, quantum wells, and quantum dots, down to the nanometer level, corresponding to the thickness of few monolayers and even less (i.e., in the sub-nm range). Technological methods for growing heterostructures are constantly being improved, and along with the study of self-organized heterostructures (such as quantum dots), methods are being developed for obtaining structures with an ordered complex surface topology. An important role in achieving the desired properties of heterostructures and developing new technological methods is played by the development of various analytical methods for studying the structural, optical, and electrophysical properties of quantum-sized heterostructures. Seven articles of this Special Issue reflect almost all of the above areas of research on semiconductor heterostructures.

In this Special Issue, Davydov et al. [1] described experimental and theoretical studies of phonon modes in GaN/AlN superlattices grown by plasma-assisted molecular beam epitaxy with a period varying from 4 to 9 atomic layers (monolayers). Using a detailed group theoretical analysis, the origin of phonon modes in GaN/AlN superlattices from the modes of bulk GaN and AlN crystals was established. Further ab initio calculations within the framework of the density functional theory made it possible to calculate the phonon states in GaN/AlN superlattices with both equal and unequal layer thicknesses. As a result, the frequencies of vibrational modes and the patterns of atomic displacements in superlattices were determined. Moreover, the Raman spectra of the GaN/AlN superlattices were calculated, which showed good agreement between acoustic and optical phonons, as well as the experimental measurements of a series of samples with various superlattices. These results open up new possibilities for analyzing the structural properties of GaN/AlN superlattices using Raman spectroscopy.

In the next article, Ahn et al. [2] considered the correlation between the optical localization state and the electrical deep-level defect state in an In_0.52_Al_0.48_As/In_0.53_Ga_0.47_N quantum well structure with two quantum-confined electron states and two hole sub-bands. The properties of this structure were studied by measuring photoluminescence spectra in a wide temperature range (10–300 K). The authors determined the energy position of localized states in such quantum wells using phenomenological analysis based on experimental measurements of the Fermi edge singularity and its analysis in terms of the line shape model with key physical parameters, such as the Fermi energy, the hole localization energy, and the band-to-band transition amplitude.

Measurements of the magneto-photoluminescence spectra of single-electron quasi two-dimensional InP/GaInP_2_ islands were used by Mintairov et al. [3] to find the magnetic field dispersion of the different single-particle states in the magnetic field range of 0–10 T. The measured dispersion explains the microscopic mechanism of vortex attachment in the composite fermion theory of the fractional quantum Hall effect. This allowed it to be described it in terms of the self-localization of magneto-electron states, representing progress towards the goal of engineering any properties for fault-tolerant topological quantum gates.

The following articles of the Special Issue are devoted to solving various technological problems in the implementation of various semiconductor heterostructures for light-emitting devices and transistors.

Novikov et al. [4] described a new approach to improve the efficiency of light-emitting Ge(Si) quantum dots grown using molecular beam epitaxy due to their spatial ordering on pit-patterned silicon-on-insulator substrates. As a result of this approach, spatially ordered quantum dots can be grown and photonic crystals can be formed with embedded quantum dots. With the help of theoretical and experimental studies, the optimal parameters of the pits were determined, including their period, shape, depth, etc., which provide a significant increase in the luminescence intensity of quantum dots due to the effective interaction between the output radiation and the photonic crystal modes.

The problems of selective growth of InGaAs quantum wells on GaAs substrates by metalorganic chemical vapor deposition were analyzed by Shamakhov et al. [5]. They compared the photoluminescence spectra of InGaAs/GaAs quantum wells along windows 100 µm wide. These measurements revealed a blueshift of 14(19) meV for 80(300 K) in the photoluminescence peak position when the measurement point shifted from the edge to the center of the window. This effect was explained by a local change in the growth rate of InGaAs quantum well. Moreover, the study by atomic force microscopy showed that the structures grown using standard (planar) epitaxy and selective area epitaxy have different surface morphologies, corresponding to a step-flow growth mode with a homogeneous distribution of a small step height (1.5 mL) and step-bunching growth mode, respectively. The structures grown in the latter mode demonstrated a strong variation in the surface morphology in an ultra-wide window from its center to the edge, which was explained by a change in the local misorientation of the layer due to a local change in the growth rate over the width of the windows.

Dai et al. [6] described a solution to the problem of obtaining a high concentration of holes in p-GaN/AlGaN heterostructures grown on Si substrates using metal organic chemical vapor deposition to fabricate an enhanced-mode GaN HEMT. Hall measurements showed that the optimal CpMg flow rate of 450 sccm provides a maximum doping efficiency of 2.22%. This effect was explained by the increased efficiency of the incorporation of Mg atoms into substituting Ga positions and by the positive effect of compressive stresses in this heterostructure, which restrict the development of self-compensation processes. In addition, a further improvement in the activation rate was demonstrated after adding an AlN intermediate layer (IL) to the p-GaN/AlN-IL/AlGaN structure, which was associated with effective suppression of the diffusion of Mg atoms. As a result, a high hole concentration of about 1.3 × 10^18^ cm^−3^ can be achieved in the p-GaN/AlN-IL/AlGaN structure.

In the last article of this Special Issue [7], the authors demonstrated the possibility of plasma-assisted molecular beam epitaxy to fabricate multiple GaN/AlN quantum wells (MQWs) with heterostructures that can be used in high-power ultraviolet (UV) emitters pumped by an electron beam. The main feature of these heterostructures, which can emit in the sub-250 nm UVC spectral range, is the ultra-thin quantum wells with a nominal thickness of less than 2 mL. It is important that, by optimizing the growth conditions, it is possible to grow relatively thick (up to 2 microns) stress-free GaN/AlN MQW structures with up to 400 periods. As a result, the authors demonstrated the ability of these MQW heterostructures to emit an output UV power of up to 11.8 W at a wavelength of 240 nm upon pulsed e-beam pumping with an electron beam current of 450 mA and an energy of 12.5 keV.

In general, the Special Issue includes seven articles from different groups devoted to the study of the basic properties and technological peculiarities of various semiconductor heterostructures, including quantum wells, quantum dots, and superlattices, grown by metal–organic vapor deposition or molecular beam epitaxy. These structures are designed to implement various devices, such as InGaAs semiconductor lasers, quantum electronics devices, high-power UVC emitters with electron beam pumping, Ge(Si) infra-red emitters, and various types of HEMT.

## References

[1] Davydov V.Y., Roginskii E.M., Kitaev Y.E., Smirnov A.N., Eliseyev I.A., Nechaev D.V., Jmerik V.N., Smirnov M.B. (2021). Phonons in Short-Period GaN/AlN Superlattices: Group-Theoretical Analysis, Ab initio Calculations, and Raman Spectra. Nanomaterials.

[2] Ahn I.-H., Kim D.Y., Lee S. (2021). Correlation between Optical Localization-State and Electrical Deep-Level State in In_0.52_Al_0.48_As/In_0.53_Ga_0.47_As Quantum Well Structure. Nanomaterials.

[3] Mintairov A.M., Lebedev D.V., Vlasov A.S., Bogdanov A.A., Ramezanpour S., Blundell S.A. (2021). Fractional Charge States in the Magneto-Photoluminescence Spectra of Single-Electron InP/GaInP2 Quantum Dots. Nanomaterials.

[4] Novikov A.V., Smagina Z.V., Stepikhova M.V., Zinovyev V.A., Rudin S.A., Dyakov S.A., Rodyakina E.E., Nenashev A.V., Sergeev S.M., Peretokin A.V. (2021). One-Stage Formation of Two-Dimensional Photonic Crystal and Spatially Ordered Arrays of Self-Assembled Ge(Si) Nanoislands on Pit-Patterned Silicon-On-Insulator Substrate. Nanomaterials.

[5] Shamakhov V.V., Nikolaev D.N., Slipchenko S.O., Fomin E.V., Smirnov A.N., Eliseyev I.A., Pikhtin N.A., Kop’ev P.S. (2021). Surface Nanostructuring during Selective Area Epitaxy of Heterostructures with InGaAs QWs in the Ultra-Wide Windows. Nanomaterials.

[6] Dai J.J., Mai T.T., Wu S.K., Peng J.R., Liu C.W., Wen H.C., Chou W.C., Ho H.C., Wang W.F. (2021). High Hole Concentration and Diffusion Suppression of Heavily Mg-Doped p-GaN for Application in Enhanced-Mode GaN HEMT. Nanomaterials.

[7] Jmerik V.N., Nechaev D.V., Orekhova K.N., Prasolov N.D., Kozlovsky V.I., Sviridov D.E., Zverev M.M., Gamov N.A., Grieger L., Wang Y. (2021). Monolayer-Scale GaN/AlN Multiple Quantum Wells for High Power e-Beam Pumped UV-Emitters in the 240–270 nm Spectral Range. Nanomaterials.

